# Nrf2 activation: a key mechanism in stem cell exosomes-mediated therapies

**DOI:** 10.1186/s11658-024-00551-3

**Published:** 2024-03-02

**Authors:** Zeinab Vahidinia, Abolfazl Azami Tameh, Shirin Barati, Melika Izadpanah, Elahe Seyed Hosseini

**Affiliations:** 1 Anatomical Sciences Research Center, Institute for Basic Sciences, Kashan University of Medical Sciences, Kashan, Iran; 2 Department of Anatomy, Saveh University of Medical Sciences, Saveh, Iran; 3 Department of Anatomical Sciences, Faculty of Medicine, Tabriz University of Medical Sciences, Tabriz, Iran; 4 Gametogenesis Research Center, Institute for Basic Sciences, Kashan University of Medical Science, Kashan, Iran

**Keywords:** Nrf2, Exosomes, Stem cells, MicroRNAs

## Abstract

Exosomes are nano-sized membrane extracellular vesicles which can be released from various types of cells. Exosomes originating from inflammatory or injured cells can have detrimental effects on recipient cells, while exosomes derived from stem cells not only facilitate the repair and regeneration of damaged tissues but also inhibit inflammation and provide protective effects against various diseases, suggesting they may serve as an alternative strategy of stem cells transplantation. Exosomes have a fundamental role in communication between cells, through the transfer of proteins, bioactive lipids and nucleic acids (like miRNAs and mRNAs) between cells. This transfer significantly impacts both the physiological and pathological functions of recipient cells. Nuclear factor erythroid 2–related factor 2 (Nrf2), a transcription factor, is able to mitigate damage caused by oxidative stress and inflammation through various signaling pathways. The positive effects resulting from the activation of the Nrf2 signaling pathway in different disorders have been documented in various types of literature. Studies have confirmed that exosomes derived from stem cells could act as Nrf2 effective agonists. However, limited studies have explored the Nrf2 role in the therapeutic effects of stem cell-derived exosomes. This review provides a comprehensive overview of the existing knowledge concerning the role of Nrf2 signaling pathways in the impact exerted by stem cell exosomes in some common diseases.

**Graphical Abstract:**

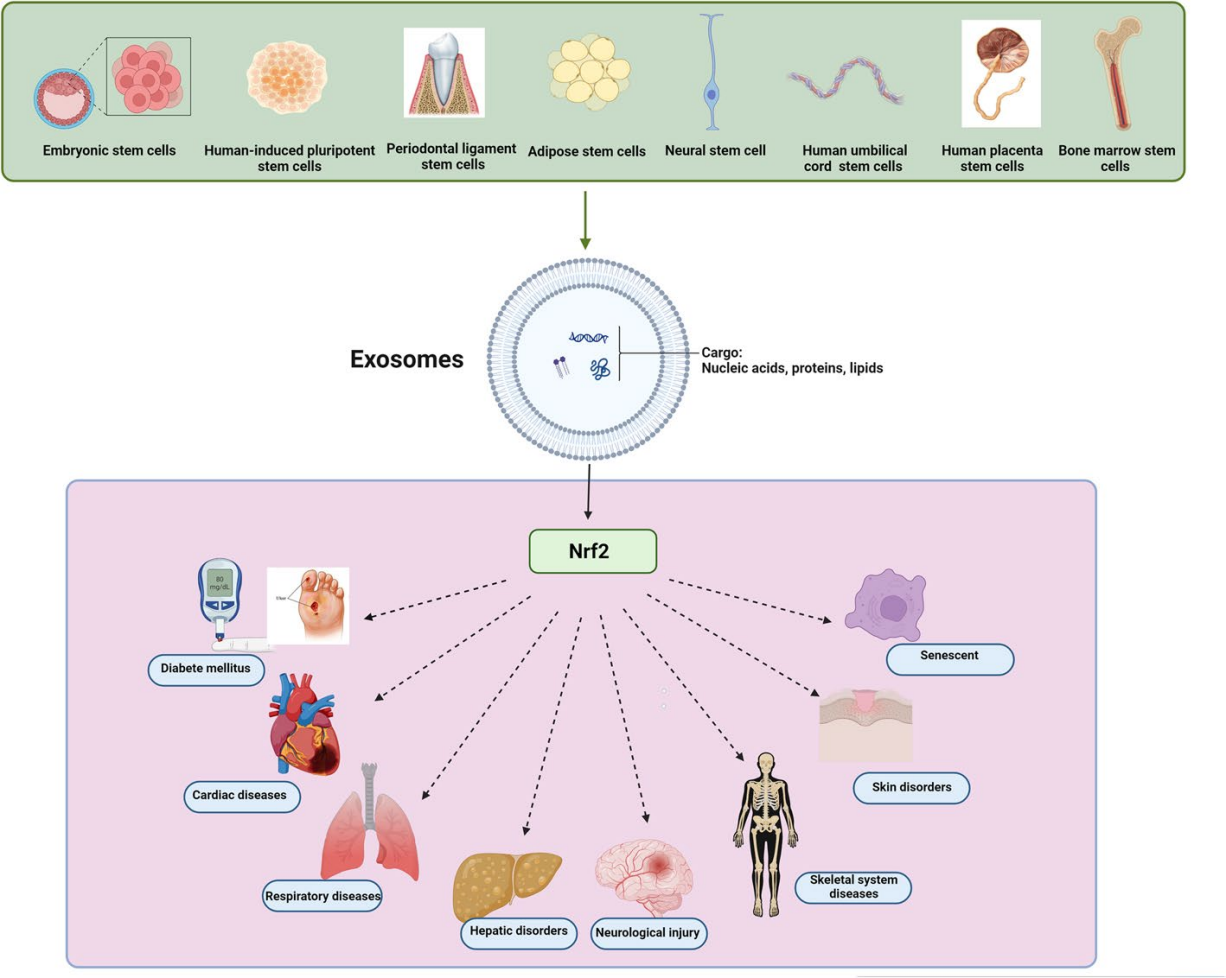

## Introduction

Stem cells are a category of cells that can self-renew and differentiate into various cell types. They are involved in a wide range of physiological and pathological processes including healing of wound, tissue regeneration and tumor formation [1]. Lately, concerns have emerged regarding the safety of using stem cells in the clinical settings. Research indicates that directly transplanting stem cells into specific tissues carries certain risks, such as low survival rates, cell dedifferentiation risk, and the potential for tumorigenesis [2]. Moreover, rejection of transplanted cells by the recipient’s body and ectopic tissue formation further restrict clinical use of stem cells in medical treatments [2]. Over the last ten years, scientists have discovered that extracellular vesicles (EVs) derived from stem cells exhibit therapeutic benefits similar to the parent cells in certain diseases [3]. EV-based therapy, compared to stem cells, provides benefits like immune silence, non-cancerous properties, excellent stability, specific homing to cells and tissues and absence of vascular blockade [4]. EVs are released by different tissues and cells and possess vesicular structures enclosed within a lipid bilayer [5]. These vesicles are categorized into exosomes (30–150 nm, obtained through ultracentrifugation at 100,000×*g*), microvesicles (100–1000 nm, collected via medium-speed centrifugation at 20,000×*g*) and apoptotic vesicles (500–5000 nm, isolated using low-speed centrifugation at 2000×*g*) [6, 7]. As consensus has not yet emerged on specific markers of EV subtypes, it is hard to distinguish exosomes or microvesicles; therefore, MSC exosomes or microvesicles are referred to as MSC-derived small extracellular vesicles (sEVs) [7–9]. At present, the main challenge for studying EVs is their isolation. Exosomes and other EVs are isolated using various methods, such as differential ultracentrifugation, ultrafiltration, polyethylene glycol-based precipitation, size-exclusion chromatography, immunoaffinity capture, or by using microfluidics [10].

The exosomes are sEVs that have the smallest average particle size, the highest homogeneity, the most complex composition, and diverse functions among all EVs. Therefore, due to these attributes, they possess the highest practical value and have undergone extensive research and widespread application [11, 12]. Exosomes comprise complex contents, such as nucleic acids including mRNAs, DNA, and noncoding RNAs, lipids, and various proteins that play a vital role in the paracrine mechanisms [13, 14]. Their bilayer lipid membrane enables them to easily penetrate cell membranes, facilitating the information transfer between cells. This ability plays a critical role in modulating the activities of target cells effectively and influencing the development of diseases [15]. Currently, it is widely accepted which the exosomes functional importance relies on their specific contents [16, 17]. These constituents consist of proteins, cytokines, lipids and genetic materials. Notably, research has demonstrated that miRNAs (miRs), transported by exosomes, have substantial impacts on diverse pathological and physiological processes, such as epigenetic modification, regulation of immune system, tumor progression, body development and more [18]. Given that the major molecular components in exosomes are miRs, they play significant regulatory functions in treating diverse diseases by delivering these specific miRs to target cells [19]. Research has highlighted the promising anti-inflammatory and injury repair capabilities of exosomes derived from mesenchymal stem cells (MSCs) [20, 21]. These exosomes are extensively being explored as nanotherapeutic agents for the treatment of stroke [22], diabetes [23], cardiac diseases [24], wound healing [25], liver and kidney diseases [26, 27], autoimmune and neurodegenerative disorders [28], respiratory diseases [29], age-related diseases [30] and other diseases. While past research showed that both MSCs and MSC-derived EVs could potentially counteract oxidative damage [31], their effectiveness in inhibiting oxidative dysfunction and structural injury is not yet fully understood. In recent years, nuclear factor erythroid 2–related factor 2 (Nrf2) has received lots of attention as a promising treatment target for a wide range of disorders [32], including neurodegenerative disorders [33, 34], cancer [35], respiratory disorders [36] and cardiocerebral vascular diseases [37]. The kelch-like ECH-associated protein 1(KEAP1)- Nrf2 pathway serves as a main defense mechanism in response to oxidative stress. It controls the transcription of antioxidant genes to eliminate the possible injury resulting from oxidation and the presence of carcinogenic substances [38]. KEAP1 regulates the activity of Nrf2 by targeting it for ubiquitination and subsequent proteasomal degradation, which is a vital process for regulating responses to injury mediated by oxidative stress. In the absence of external stimuli, Nrf2 remains in an inactive state [39]. Under stress conditions, KEAP1 becomes inactive and prevents Nrf2 ubiquitination. As a result, Nrf2 accumulates in large amounts within the cell, translocates to the nucleus, and promotes secondary antioxidative responses [40]. The KEAP1 overexpression suppresses Nrf2’s transcriptional activity; Conversely, the KEAP1 absence triggers Nrf2 activation, enabling its response to oxidative stress. In other words, alterations in the interaction between Nrf2 and KEAP1 can activate the Nrf2 pathway, leading to the production of various factors like glutamate-cysteine ligase (GCL) and heme oxygenase-1 (HO-1) [39]. Thus, specific targeting of the Nrf2/HO-1 axis might offer a new therapeutic approach for managing various human diseases, such as Alzheimer’s, diabetes, hepatotoxicity and more [41–44]. Research has confirmed that exosomes derived from MSCs could act as Nrf2 effective agonists [45, 46]. In contrast to other Nrf2 agonists, MSC- exosomes provide therapeutic benefits without toxic side effects associated with drugs and they potentially possess the capability to reach cells directly [42]. Recent studies have revealed that MSCs-derived exosomes have the ability to mitigate injuries caused by oxidative stress by modulating the Nrf2 pathway and its downstream antioxidative genes [47, 48]. It also has been reported that exosomes could serve as anti-inflammatory and antioxidants agents in conditions like skin oxidation, neurological disorders and macrophage polarization through regulation of the Keap1/Nrf2 axis [20, 49]. This review aimed to assess the significance of the Nrf2 signaling pathway in the therapeutic effects of exosomes derived from stem cells for various common diseases.

## Diabetes mellitus

**Fig. 1:**
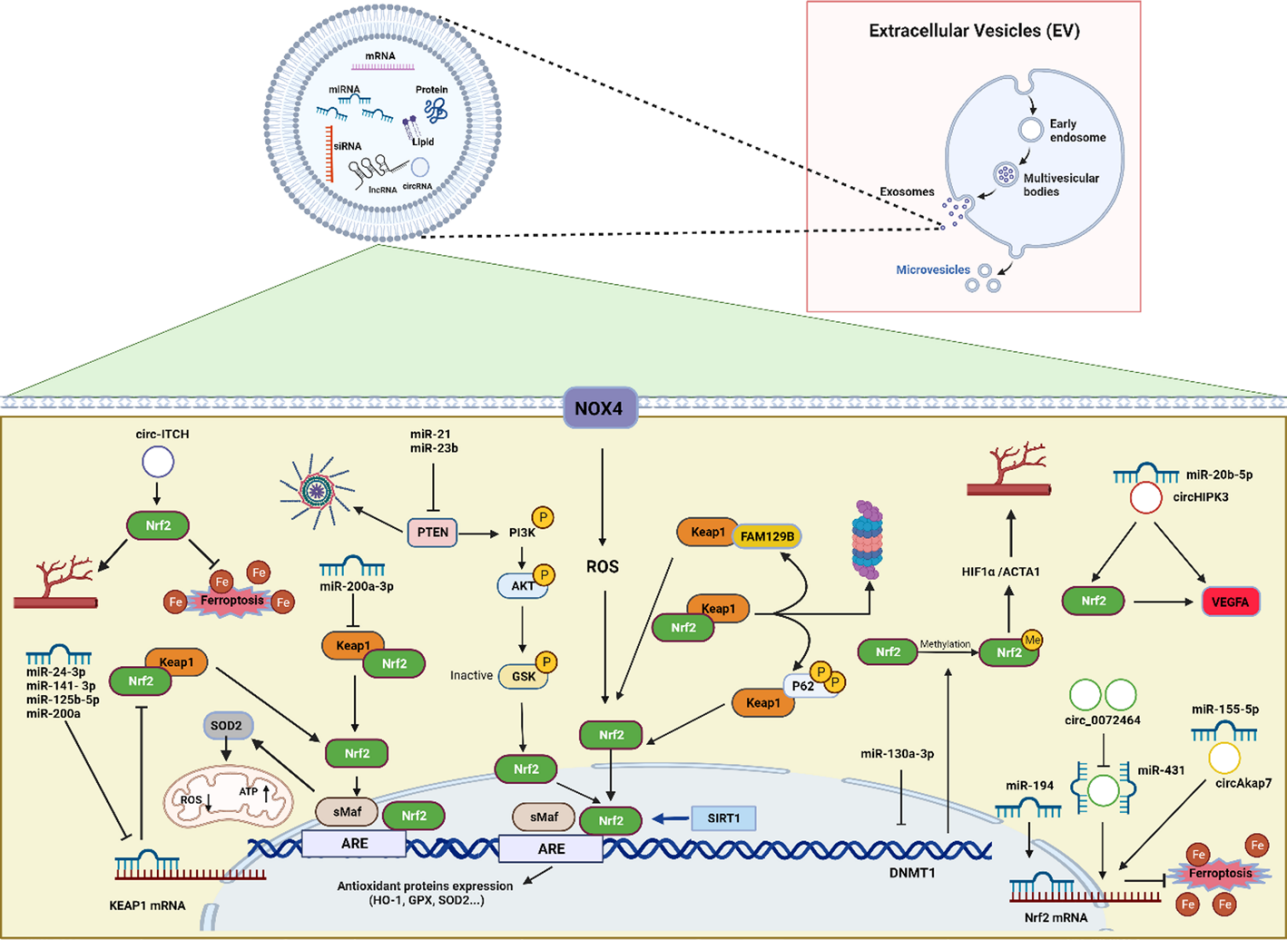
Schematic representation of effects of stem cells-derived exosomes on the Nrf2 pathway. miR-24-3p, miR-141- 3p, miR-125b-5p and miR-200a are transferred by stem cell-exosomes into recipient cells where they target Keap-1, thereby promoting Nrf2 activation. circHIPK3 released from exosomes acts as a ceRNA to bind to miR-20b-5p, which directly inhibits miR-20b-5p and upregulates Nrf2 or/and VEGFA expression, promoting angiogenesis. miR-200a-3p leads to the downregulation of Keap1, nuclear translocation of Nrf2 and promotion of SOD2 expression, resulting in high ATP production and protection against mitochondrial fragmentation. Exosomal miR-23b and miR-21 can alleviate oxidative stress, leading to a reduction in neuroinflammation and providing neuroprotective effects by PTEN/PI3K/AKT/Nrf2 pathway. ADSCs-exosomes could attenuate inflammation and oxidative stress induced by high glucose in podocytes through the upregulation of FAM129B and reactivation of the Nrf2-HO-1 pathway, FAM129B competes with Nrf2 and binds to Keap1, leading to reduced Nrf2 ubiquitination and thereby activation of the Nrf2 pathway. MSCs-exosomes are able to protect against acute liver injury through activation of the P62-Keap1-Nrf2 pathway. P62 serves as an important regulator located upstream of the Keap1-Nrf2 pathway. In response to oxidative stress, P62 competitively interacts with the Nrf2-binding site of Keap1 and inhibits the Nrf2 ubiquitination. It has been demonstrated that miR-100-5p-enriched exosomes have capability to decrease oxidative stress through the regulation of the Nox4-ROS-Nrf2 axis. miR-130a-3p suppresses Nrf2 methylation and upregulates Nrf2 expression through DNMT1 inhibition to activate HIF1α/ACTA1 axis, thereby improving angiogenesis**.** In addition, exosomes are able to upregulate the expression level of SIRT1 which its restoration results in an increase in Nrf2 and HO-1. Exosomal circ-ITCH suppresses ferroptosis and enhances the angiogenesis by the Nrf2 activation. circ_0072464 shuttled by BMSC-derived EVs can reduce ferroptosis through miR-431 inhibition and the subsequent increase in miR-431-mediated Nrf2 expression. Exosomes loaded with miR-194 alleviate damage caused by ischemia by increasing the Nrf2/HO-1 activation, leading to the downregulation of ferroptosis. Exosome-circAkap7 lessens oxidative stress against ischemic damage via increasing nuclear transcription of Nrf2 by absorbing miR-155-5p. ACTA1, Skeletal muscle actin alpha 1; ceRNAs, competing endogenous RNAs; DNMT1, DNA methyltransferase 1; DPN, Diabetic peripheral neuropathy; HIF1α, Hypoxia inducible factor 1 subunit alpha; HO-1, heme oxygenase-1; KEAP1, Kelch-like ECH-associated protein 1; MSCs, mesenchymal stem cells; Nox4, NADPH oxidase 4; Nrf2, factor nuclear factor-erythroid 2-related factor 2; PI3K, The phosphoinositide 3-kinase; PTEN, Phosphatase and tensin homolog deleted on chromosome 10; SIRT1, silent information regulator 1; SOD; Superoxide dismutase, VEGFA, vascular endothelial growth factor-A

Diabetes is a heterogeneous medical condition marked by high blood sugar (glucose) levels or hyperglycemia due to impairment of insulin secretion or defects in the action of insulin or a combination of both factors [50]. Diabetic nephropathy stands out as a prevalent complication of diabetes and is considered as the main factor leading to end-stage kidney disease [51]. Approximately one-third of individuals with diabetes experience kidney dysfunction, resulting in a poor prognosis and substantial long-term social and financial burden [52]. The primary approach for treating diabetic nephropathy involves managing symptoms. This includes methods like regulating blood sugar levels, managing blood pressure and decreasing proteinuria [53]. At present, effective therapeutic medications designed specifically for diabetic nephropathy are still lacking [52]. Consequently, exosomes have the potential to function as biomarkers and as therapeutic agents for a range of kidney disorders [54]. Growing interest has been shown in the therapeutic potential of adipose-derived stem cells (ADSCs)-exosomes for diabetic nephropathy treatment in recent years [55]. These exosomes have the ability to improve podocyte injury induced by high glucose and slow down diabetic nephropathy progression [56]. Furthermore, it has been demonstrated that Nrf2 has therapeutic effects on diabetic nephropathy [57]. Liu et al. reported that Nrf2^-/-^ mice exhibited more severe diabetic kidney disease than wild type [58]. Moreover, individuals with Nrf2 genetic mutations are at an increased risk of complications related to diabetes, such as nephropathy, retinopathy, peripheral neuropathy, foot ulcers and microangiopathy [59]. It has been revealed that ADSCs-exosomes could attenuate inflammation and oxidative stress caused by high glucose in podocytes. This effect occurs through the upregulation of FAM129B and reactivation of the Nrf2-HO-1 pathway, offering a novel approach for clinical treatment [60]. FAM129B is a protein known for its antioxidant properties and has the ability to prevent apoptosis in tumors. It competes with Nrf2 and binds to Keap1, leading to reduced Nrf2 ubiquitination and activation of the Nrf2 pathway (Fig. 1) [61, 62].

Diabetic peripheral neuropathy (DPN) is known as the most common form of neuropathy in world and its prevalence rate rises with the prolonged duration of diabetes over time [63]. DPN is a common complication related to both type 1 and type 2 diabetes. This condition results in disabling neuropathic pain and in severe cases, lower extremity amputation and imposes a substantial economic burden on society [64]. Research has indicated the pivotal role of Nrf2 in various pathways that impact the development of diabetic neuropathy [65]. Furthermore, Nrf2 is involved in regulating cellular redox homeostasis, inflammatory responses and improving mitochondrial function, all of which contribute to its neuroprotective effects in DPN [66]. Recent findings provided evidence demonstrating that delivering miR-130a-3p via EVs derived from ADSC is able to activate Nrf2/hypoxia inducible factor 1 subunit alpha (HIF1α)/ skeletal muscle actin alpha 1 (ACTA1) axis. This activation occurs by inhibiting DNA methyltransferase 1 (DNMT1) to improve DPN through the suppression of apoptosis in schwann cells (Fig. 1) [67]. Furthermore, another study has demonstrated that Nrf2 overexpression in schwann cells contributes to the repair of peripheral nerve injury in DPN [68]

Diabetic foot ulcers (DFU) have serious implications, often resulting in high mortality and morbidity rates [69], along with imposing a substantial socioeconomic burden [70]. Previous research has indicated that DFU development is influenced by multiple factors such as peripheral arterial disease, neuropathy and infections [71]. The wound healing process is marked by numerous events, such as inflammation, angiogenesis and the extracellular matrix (ECM) remodeling. As a result, the pathophysiology of DFU is extraordinarily complicated [72]. Patients' hyperglycemic state set off DFU pathophysiological hallmarks, which include abnormal vascular development, neuropathy, and immunological response [73]. Hyperglycaemia can lead to dysfunction in vascular endothelial cells by elevated plasma thromboxane A2 and reduced levels of vasodilators. This condition results in ischemia and ulcers. Therefore, it is crucial to regulate blood vessel formation and integrity to prevent the DFU development [74]. In hyperglycemic conditions, the dysfunction and senescence of endothelial progenitor cells (EPCs) result in higher levels of reactive oxygen species (ROS) and hinder DFU healing [75]. EPCs with their capacity to differentiate directly into endothelial cells, have a vital role in the process of revascularization during wound healing [76, 77]. Diabetic mice with Nrf2 knockout compared with the diabetic wild-type littermates exhibited delayed wound closure along with increased oxidative DNA injury and apoptosis [78]. It also has been demonstrated that overexpression of Nrf2 is able to protect diabetic EPCs against dysfunction induced by oxidative stress in vitro [79]. Besides, Nrf2 activation pharmaceutically improves the function of EPCs and accelerates diabetic wound healing in rodents [80, 81]. Loss of Nrf2/ARE activity contributes to increased oxidative stress, that could worsen the impaired function of endothelial cells and abnormal angiogenesis which typically observed in diabetes [82]. Studies have revealed the regulatory Nrf2 role in the regulation of vascular endothelial growth factor-A (VEGFA) [83]. Evidence from an earlier study showed that the Nrf2 has capability of regulation of endothelial proliferation and vascular growth through mechanisms dependent on VEGFA [84]. Increased levels of Nrf2 and VEGFA provide protection against injuries related to diabetic foot ulcers. This protection is achieved by inhibiting oxidative stress and inflammaion [85] and promoting microvascular formation [86]. Human ASC-exosomes were found to mitigate premature senescence in EPCs triggered by high glucose levels. Additionally, these exosomes improved wound healing in diabetic rats [87]. In this research, overexpressed Nrf2 of ASC-exosome led to a significant reduction in ulcer size and granulation tissue formation. This effect was accompanied by an elevated level of growth factor expression and improved angiogenesis achieved through the modulation of various proteins [87].

CircHIPK3 is a type of non-coding RNA that operates through different types of mechanisms, such as interacting with miR, serving as a sponge for RNA binding protein, acting as templates for protein translation, or directly regulating gene expression [88]. Research has provided evidence indicating the involvement of circHIPK3 in many different physiological and pathological mechanisms [89]. circHIPK3 is considered as a crucial factor in various vascular conditions, including diabetic wounds and atherosclerosis [88, 90]. It has been reported that human umbilical cord mesenchymal stem cells (hUC-MSCs) derived circHIPK3 overexpressing exosomes are able to protect cells from injuries induced by high glucose and boost angiogenesis in diabetic wounds. This protective impact is likely mediated through the direct inhibition of miR-20b-5p activity which leads to elevated expressions of Nrf2 and VEGFA. As a result, targeting the circHIPK3/miR-20b-5p/Nrf2/VEGFA axis could be a novel approach for the DFU treatment (Fig. 1) [74]. It has also been demonstrated that exosomal circRNA-itchy E3 ubiquitin protein ligase (circ-ITCH) derived from bone marrow- derived stem cell (BMSCs) suppressed ferroptosis and enhanced the human umbilical vein endothelial cells (HUVECs) angiogenesis by the Nrf2 pathway activation through the recruitment of TATA-Box-binding protein associated factor 15 (TAF15) protein. As a result, this mechanism accelerated the DFU wound healing (Fig. 1) [91]. In addition, in the study by Wang et al., exosomes produced by BMSC were found to enhance processes such as EPC tube formation, neovascularization, collagen deposition and re-epithelialization. Notably, these positive effects were inhibited in the absence of Nrf2. It has also been reported that positive effects of these exosomes were ampilified when treated with a combination of exosomes derived by BMSC and tert-butylhydroquinone (tBHQ), a small-molecule activator of Nrf2. These findings indicate that using Nrf2 activators in conjunction with BMSC-exosomes could present a novel approach for treating chronic diabetic wounds [45].

## Skeletal system diseases

Osteoporosis and intervertebral disc degeneration (IDD) are prevalent conditions, both among younger and elderly population which significantly affect life quality [92, 93]. Osteoporosis or low bone mineral density affects about 44 million Americans, which accounts for 55% of people 50 years of age and older [94]. Osteoporosis can cause bones to become more fragile, making them susceptible to low-energy fractures. Usually, these fractures happen in regions of the skeleton with high trabecular content and weight-bearing condition such as hip, spine and the wrist [95, 96]. Oxidative stress play a role in pathophysiology of osteoporosis and can lead to significant cellular injury, including cell apoptosis, necrosis and autophagy [97, 98]. Additionally, overproduction of ROS can trigger disruption of mineral tissue homeostasis and the remodeling process of bone by inducing oxidative stress [99, 100]. Studies have shown the key role of Nrf2 in osteoporosis. According to recent studies, Nrf2 plays an significant role in bone tissue homeostasis and its activation can triger antioxidant responses against ROS, thus regulating osteoporosis occurrence [101, 102]. Exosomes derived from MSC have been demonstrated to improve bone loss in animal models of bone defects like osteoporosis and osteonecrosis [103, 104]. ADSCs-exosomes have been shown to reduce ROS accumulation and mitochondria dysfunction in osteoblasts. Pretreatment with ADSCs-exosomes could induce Nrf2 expression in dexamethasone -stimulated osteoblasts showing that these exosome insert their anti-apoptotic effects via Nrf2. Thus, ADSCs-exosomes could reduce apoptosis and oxidative stress by modulating Nrf2/HO-1 expression following dexamethasone exposure and prevent the progression of glucocorticoid-induced osteoporosis in vivo [11].

IDD is known by serious spinal symptoms, such as low back pain and sciatica syndrome [105] which is resulted from degenerative disc conditions [106]. Treatment approaches for IDD include ECM generation and pro-anabolic treatment via regulating ROS microenvironment [107]. ROS functions as an important factor in the IDD progression because of its’ degenerative environment [108] and exosomes are able to inhibit the elevated ROS by the activation of NLR family pyrin domain containing 3 (NLRP3) inflammasome [109]. According to reports, Nrf2 expression level is low in the nucleus pulposus of degenerated disk [110] and activation of Nrf2 in the region could alleviate IDD progression through mobilizing the intrinsic antioxidant capacity of cells and cosequenltly reduction of excessive ROS [111–113]. BMSC-derived exosomes have the potential to alleviate IDD process through regulating Keap1/Nrf2 pathway which results in a reduction in excessive levels of ROS [114]. Current research findings show that the occurrence of ferroptosis in nucleus pulposus cells is closely linked to IDD pathogenesis, suggesting that targeting ferroptosis with the inhibition of lipid peroxidation via Nrf2 overexpression could be a promising and novel approach for the treatment of IDD [115, 116]. It has been demonstrated that circ_0072464 shuttled by BMSC-derived EVs can reduce ferroptosis in nucleus pulposus cells by miR-431 inhibition and the subsequent increase in miR-431-mediated Nrf2 expression in both vitro and in vivo models. According to these results, circ_0072464 could serve as a promising therapeutic option for the IDD treatment (Fig. 1) [115].

## Respiratory diseases

Acute lung injury (ALI) and acute respiratory distress syndrome (ARDS) are known as common life-threatening lung diseases and syndromes of severe respiratory failure with substantial morbidity and mortality rates in critically ill patients [117, 118]. ALI is marked by extensive inflammation within the lungs, along with the accumulation of inflammatory cells especially macrophages and neutrophils. This accumulation causes injury to both lung endothelial cells and epithelial barriers [119]. Individuals with pneumonia or sepsis are highly vulnerable to ALI, indicating a direct association with infection [120]. Cell-based therapies like the administration of human amniotic mesenchymal stem cells (hAMSCs) can have positive outcomes in ALI treatment and provide protection against lung inflammation induced by lipopolysaccharides (LPS) [119, 121, 122]. However, the therapeutic effects of stem cells-based treatment pose challenges due to complex procedures involved in isolating, purifying, and storing these cells in a sterile manner. Additionally, inflammatory environments do not support the transplanted stem cells survival. Therefore, the use of sEVs could serve as an alternative treatment option to mimic the beneficial effects of stem cells [123] and have the ability to provide protection in a wide range of inflammatory diseases [124]. It has been found that activation of Nrf2 confers protection against LPS-induced lung injury [125] and its’ overexpression could mitigate the injury [122].

Therefore, modulation of Nrf2 expression could offer a new method for treating ALI. It is also reported that Nrf2 could slow down lung injury development through the activation of antioxidant genes and modulation of NLRP3 inflammasome in lung inflammation in vitro model induced by lipopolysaccharides (LPS) [125, 126].

Macrophages and pulmonary microvascular endothelial cells (PMVECs) are the most important mediators and victims of ALI [127]. When a pathogen invades, alveolar macrophages become activated and shift to a M1 phenotype (pro-inflammatory). These activated alveolar macrophages release inflammatory cytokines like interleukin 1 (IL-1), tumor necrosis factor-α (TNF-α) and interleukin 6 (IL-6) [128, 129]. Overproduction of inflammatory cytokines and ROS elevation leads to membrane and DNA damage of PMVECs [130]. Shen et al. showed that ADSCs exosomes have the ability to regulate Keap1/Nrf2 pathway within macrophages in lung tissues. This regulatory process ultimately results in a protective effect against lung injury induced by cecal ligation and puncture [49]. Nrf2 regulates numerous cytoprotective genes like glutathione Peroxidase 4 (GPX4) [116]. The antioxidant enzyme GPX4 functions as a negative regulator of ferroptosis through diminishing lipid peroxidation [131, 132]. Studies have revealed that the delivery of miR-125b-5p through exosomes derived from ADSCs can attenuate PMVECs ferroptosis induced by inflammation during sepsis-induced ALI through the regulation of Keap1/Nrf2/GPX4 expression, therefore improve the ALI (Fig. 1) [133]. BMSC-exosomes have been shown to decrease ALI related in cardiopulmonary bypass by mitigating inflammatory responces and oxidative stress. The mechanism behind this effect likely involves nuclear factor kappa B (NF-κB) p65 pathway as well as the protein kinase B (Akt)/Nrf2/HO-1 signaling pathways [29]. It was found that hUC-MSCs‐derived exosomes play a pivotal role in promoting Nrf2 expression and nuclear translocation. This effect is mediated through the miR-199a-5p transduction and its targeted binding with caveolin1. Consequently, this process increases the antioxidant enzyme expression within the cells of the lung, effectively regulating oxidative stress induced by sulfur mustard. Sulfur mustard is a chemical warfare agent known for the production of blister formation and can result in a series of systemic damages, particularly severe ALI [134].

Growing research has revealed that BMSCs possess biological properties that modulate the immune system and offer therapeutic benefits in alleviating ARDS, mainly through the secretion of their exosomes [135, 136]. BMSCs-exosomes have the ability to produce beneficial outcomes, such as enhancing the epithelial barrier repair in pulmonary alveoli [134]. They could prevent apoptosis induced by hyperoxia in type II alveolar epithelial cells (AECIIs) [137]. AECIIs are lung epithelium stem cells which have essential secretory and regenerative functions and maintain lung homeostasis [138]. Various studies have shown that the AECIIs apoptosis plays a crucial role in the development of sepsis-induced ARDS [139]. It has been described that BMSC-exosomes effectively reduce sepsis-caused apoptosis in AECIIs and consequently ARDS by recovering the mitochondrial dysfunction mediated by Nrf2 [140].

## Cardiac diseases

Myocardial infarction (MI) occurs due to a sudden decrease or cessation of blood flow in a coronary artery which leads to severe and prolonged acute ischemia in a specific area of the heart muscle, ultimately resulting in myocardial necrosis [141, 142]. MI, known as the most common cardiovascular disease, is a serious threat to life and health [143]. There is growing evidence that BMSCs transplantation can be considered as a promising treatment for MI due to their anti-inflammatory, anti-fibrosis and angiogenic properties [144]. However, animal models and clinical trials have discovered the limited effectiveness of BMSCs for MI that may be attributed to the poor local microenvironment and high levels of inflammation reactions within ischemic tissue, leading to engrafted cells death following administration of BMSCs [145, 146].

It has been demonstrated that the MSCs paracrine effect promotes angiogenesis, myocardial tissue repair, immunosuppression and stem cell homing through secretion of EVs, transforming growth factor beta T helper 1 (TGF-β), TNF-a, VEGF and other growth factors and cytokines [147]. ADSC-derived Exosomes could promote angiogenesis in the ischemic region and protect cardiac myocytes from excessive oxidative stress in the infarct area [148–150]. Additionally, exosomes have the potential to suppress inflammation and improve cardiac function and fibrosis in an animal model of MI [151]. Oxidative stress serves as a crucial mechanism in myocardial damage following MI [143]. An imbalance between the ROS production and their removal by body's antioxidant mechanisms leads to the macromolecules damage and redox signaling interruption that affect structure and function of heart, leading to myocardial hypertrophy, impaired contractile function, and fibrosis seen in chronic heart failure [152]. The Nrf2/ARE axis act as key signaling molecules in preventing oxidative damage of cardiac cell [153] and protects the heart against cardiac dysfunction and maladaptive remodeling [154, 155]. Previous studies demonstrated that Nrf2 signaling is involved in the onset and progression of numerous heart disorders, including MI, myocarditis and atrial fibrillation [156, 157]. Nrf2/HO-1 activation upregulates the transcription of several endogenous antioxidants and protects cardiomyocytes from damage caused by oxygen radicals [158]. Exosomes derived from fibronectin type III domain-containing protein 5 (FNDC5)-preconditioned BMSCs play a protective role against MI through anti-inflammatory effects and macrophage polarization. These effects are mediated by the NF-κB signaling pathway and the Nrf2/HO-1 axis [46]. Chen et al. reported that exosome isolated from human-induced pluripotent stem cells (iPSCs) -derived MSCs could increase the survival of cardiomyocytes, improve cardiac function, reduce the extent of heart tissue damage and suppress oxidative stress. However, these positive effects of exosomes were notably reversed when LY294002, an inhibitor of the Akt/Nrf2/HO-1 pathway, was used. This suggests that exosomes could potentially improve MI triggered by severe acute pancreatitis by activating the Akt/Nrf2/HO-1 pathway [159]. The myocardial tissue of rats with atrial fibrillation exhibited reduced levels of Nrf2 and HO-1. It was revealed that Nrf2-overexpressing BMSC-exosomes could inhibit arrhythmias caused by atrial fibrillation, myocardial fibrosis, inflammation and apoptosis by activating the Nrf2/HO-1 axis [24]. Moreover, exosomes derived from silent information regulator 2 homolog 1 (Sirt1)-overexpressing ADSCs could contribute to tube formation, cell migration, and the recruitment of endothelial progenitor cells (EPCs) to the site of repair. This process facilitates the repair of the injured cardiac area through the Nrf2/CXCL12/CXCR7 pathway [160]. Sirt1 is a deacetylase dependent protein on nicotinamide adenine dinucleotide (NAD +) which regulates acetylation of specific transcription factors such as Nrf2 to prevent damage caused by oxidative stress and inflammation [161, 162].

## Liver diseases

Acute liver injury, a serious metabolic dysfunction, results from significant damage to hepatic cells and is frequently observed in various severe liver diseases [163]. The application of MSCs-exosome has shown positive results in different exprimental models of liver diseases, such as liver fibrosis, hepatocellular carcinoma and drug-induced acute liver injury [164–166]. Researchers observed that glutathione peroxidase 1 (GPX1) derived from hUC-MSCs exosomes effectively lower the levels of ROS and malondialdehyde (MDA) in liver and inhibit apoptosis induced by oxidative stress in liver failure [167]. In addition, ADSCs-derived exosomes could also alleviate ROS and MDA contents in the liver and enhance the activity of superoxide dismutase (SOD) in hepatic ischemia–reperfusion (I/R) injury [168]. Therefore, exosomes derived from MSC can be considered as an effective therapeutic intervention for liver diseases. p62-Keap1-Nrf2 pathway serves as a crucial controller in minimizing iron toxicity in hepatocellular carcinoma cells through the activation of gene transcription related to iron and ROS metabolism [169]. P62 is a key upstream regulator of the Keap1-Nrf2 axis which competitively interacts with Nrf2-binding site of Keap1 and inhibits Nrf2 ubiquitination, thereby stimulating the transcription of downstream genes [170]. P62-Keap1- Nrf2 signaling pathway plays a key role in the maintenance of redox homeostasis [171]. It has been documented that pretreated MSCs-exosome elicits better transplantation and therapeutic efficacy. A recent study found that exosomes derived from baicalin-pretreated MSCs are able to protect against acute liver injury induced by the D-galactosamine and lipopolysaccharide (D-GaIN/LPS) through activation of the P62-Keap1- Nrf2 pathway (Fig. 1) [172].

Non-alcoholic fatty liver disease (NAFLD) is a chronic liver disease that [173] is linked to metabolic disorders, like insulin resistance, obesity and type 2 diabetes [174]. NAFLD has the potential to advance into non-alcoholic steatohepatitis (NASH) and, over time, develop into cirrhosis [175]. MSCs-exosomes in fatty liver have been reported to be hepatoprotective by lipid metabolism regulation and modulation of oxidative stress and inflammation [26, 176]. Keap-1 deletion results in the enhancement of Nrf2 nuclear translocation which is followed by Ho-1 gene expression which finally results in reduction of the ROS generation [177]. Additionally, suppression of Keap-1 was shown to reduce inflammasome activation induced by metabolic stress, oxidative stress and impaired lipid metabolism in the hepatocytes [178]. It has been demonstrated that exosomal miR-24-3p derived from MSCs by targeting Keap-1 in hepatocytes treated with palmitate, could inhibit the fatty acid synthesis and NF-kB signaling pathway and improve activation of Nrf2 axis, thereby exerting a therapeutic potential against NAFLD (Fig. 1) [179].

NASH, a more severe subtype of NAFLD, is identified by steatosis, ballooning degeneration of hepatocytes, inflammation and the hepatocytes fibrosis [180]. This is a progressive disorder that can eventually cause cancer and cirrhosis of the liver [181]. Oxidative stress has been characterized as an important factor in the progression of NASH [182, 183]. Research exhibited that EVs derived from human liver stem cells could decrease hepatic TNF-α and IL-1β levels in animals with NASH [184]. Kang et al. demonstrated key role of Nrf2 signaling pathway in the treatment of NASH by stem cell derived exosomes. They reported that hUC-MSCs exosomes provide positive therapeutic effects including anti-lipid deposition, anti-oxidative stress and anti-inflammatory through Nrf2/NQO-1 pathway in NASH experimental model [185].

## Neurological injury

Neurological diseases refer to a wide range disorders affecting the central and peripheral nervous systems which make them the leading cause of disease burden world wide [186, 187]. However, the available and approved treatment options for these conditions are limited in comparison to other injured areas in the body [188, 189]. Exosomes have been shown to have a significant impact on treating neurodegenerative diseases, nerve injuries, and other neurological disorders [190]. Despite earlier studies suggesting that MSCs and EVs derived from MSCs can suppress oxidative injury [31, 191], their effectiveness in mitigating oxidative neuronal dysfunction and structural damage is not fully understood. Exosomes insert strong antioxidant effect like GPX enzyme activation and ferroptosis inhibition [161] which could ameliorate ROS induced neuronal injury [192]. In the research conducted by Li X et al. it was showed which exosomal miR-194 derived from MSC exerts neuroprotection after oxygen–glucose deprivation/reoxygenation (OGD/R) -induced neuronal injury which is an in vitro ischemic stroke model [193]. Nrf2-mediated therapies exert protective effects against various neurological problems in response to oxidative stress [15, 64]. The results from in vitro and in vivo experiments reveal that exosomes derived from circAkap7-modified ADSCs, referred to as exosomal circAkap7, could exert neuroprotection against ischemic damage by enhancing ATG12 (Autophagy Related 12)-mediated autophagy and mitigate oxidative stress by activation of Nrf2 nuclear transcription (Fig. 1) [194]. In an in vitro model of hypoxia/reperfusion injury, the co-culture of neurons with EVs derived from neural stem/progenitor cells (NPCs) prevented the apoptosis of neurons via the induction of the Nrf2 nuclear translocation that in turn regulates the expression of oxidative stress-induced kinases [195]. MSC-derived exosomes containing miR-194 are able to reduce injury after OGD/R via suppressing CNC homology 1 (Bach1) expression and Nrf2/HO-1 pathway activation through the miR-194 delivery to endothelial cells of brain vessels that leads to the downregulation of ferroptosis (Fig. 1) [193]. EVs derived from human neural stem cells (hNSC) possess the ability to inhibit apoptosis induced by oxidative stress and promote axons growth and angiogensis after ischemia induced neuronal injury. They could also enhance Nrf2 translocation to the cell nucleus in order to up-regulate antioxidant enzymes which results in intracellular ROS reduction [195]. Methotrexate (MTX) ia a chemotherapeutic agent that can cause neurotoxic effects on the central nervous system (CNS) [196] and it has been reported thet exosomes from ADSCs could alleviate MTX induced neurotoxicity through the activation of the Nrf2-ARE signaling pathway [196]. In addition, it has been identified that the protective effect conferred by MSCs-exosome against cognitive deficits in aged mice linked to their ability to prevent ferroptosis in the hippocampus by the modulation of the SIRT1/Nrf2/HO1 signaling pathway [197]. MSCs-exosome can reduce early brain damage and improve cognitive impairment following subarachnoid hemorrhage. It has been demonstrated that BMSC-exosomal miR-23b could alleviate oxidative stress and pyroptosis that modulate neuroinflammation and insert neuroprotection after intracerebral hemorrhage. Phosphatase and tensin homolog deleted on chromosome 10 (PTEN) is considered as a target gene responsible for mediating the anti-inflammatory and antioxidant properties of miR-23b through modulating the Nrf2 signaling pathway and activating the NLRP3 inflammasom (Fig. 1) [198]. Exosomes derived from MSC have the wide therapeutic potential for improvement of neurological disorders which are triggered through astrocytosis by the activation of Nrf2-NF-kB signaling pathway [20]. The results suggest that miR-100-5p-enriched trophoblast stage-derived MSCs (T-MSCs) exosomes have a protective effect against the loss of dopaminergic neurons. These exosomes contribute to the maintenance of nigrostriatal system function, improvement of motor impairments, and reduction of oxidative stress by the regulation of the NADPH oxidase 4 (Nox4)-ROS-Nrf2 axis (Fig. 1) [199].

## Age-related diseases

Aging is considered as an inevitable biological process that results in progressive decline of tissue and organ function [200] and is determined by the senescent cells accumulation in numerous tissues which result in the disruption of homeostasis and decline in regenerative capacity. This phenomenon is associated with the expression of senescence-associated β-galactosidase, the cyclin-dependent kinase inhibitors P16 and P21, elevated oxidative stress levels, and other hallmarks [200, 201]. Angiogenesis plays a critical role in process of wound healing and tissue regeneration through the restoration of blood supply and the delivery of nutrients to damaged area [202]. Endothelial cells as a key element in angiogenesis, experience function impairment as they undergo senescence [203, 204] and a higher presence of these cells are present in aged tissues [203]. It has been shown that there is an age-dependent difference in wound healing process between old and young individuals because of inadequate local angiogenesis and impaired tissue repair in aged poeple [203, 205]. The therapeutic capacity of stem cells-derived exosomes for diseases related to aging is barely reported. Recently, stem cells-derived exosomes have gained great attention in a number of studies in aging-related diseases [206] because of their pro-angiogenic effects at the injury sites [207, 208]. In a recent investigation [209] it was revealed that exosomes from ADSCs have the ability to mitigate the senescence characteristics in osteoarthritic osteoblasts. Chen et al. investigated the impact of human embryonic stem cells (hESCs) ‐exosomes on HUVECs undergoing senescence triggered by D‐galactose. They discovered that chronic treatment with hESCs ‐exosomes could diminish aging indicators and restore impaired functions, including migration, proliferation and tube formation by the transfer of miR‐200a [47].

Oxidative stress is considered as a contributor to the aging process and Nrf2 signaling could be considered as a potential defense mechanism against oxidative stress by senescence control [210]. Older cells have a lower basal Nrf2 protein expression level than cells from young adults which highlightes the importance of Nrf2 activity in determining species longevity [211, 212]. Suppression of Nrf2 expression in "young" cells leads to noticeable impairment in cellular function, while enhancing Nrf2 activity was proven to effectively counteract cellular senescence and render them similar to young cell [178, 213]. Exosomes derived from MSCs were observed to significantly alleviate aging-related senescence of CD4 + T cells. This reduction in senescence was attributed to the exosomes ability to decrease oxidative damage, lower senescence-associated secretory phenotype (SASP) expression, diminish aging-related proteins such as p53, and other markers of aging. miR-21 downregulates PTEN and boosts the activation of phosphoinositide 3-kinases (PI3K) and AKT that lead to Nrf2 gene expression (Fig. 1) [214]. Pressure ulcers, especially in people who are elderly, are known to exhibit poor healing due to age-associated changes in skin tissue [202, 203]. It has been demonstrated that aging cells have higher levels of KEAP1, and it is thought that Keap1 overexpression influences activity of Nrf2 in in the elderly [178, 210]. On the other hand, the increased Nrf2 activity observed in long-lived species is attributed in part to a reduction in Keap1 expression [215]. Treatment with the hESCs-derived exosomes was shown to speed up healing of pressure ulcer and stimulate angiogenesis at sites of wound through rejuvenating endothelial cell senescence by Nrf2 activation in aged mice. This anti-aging effects occurs via the transfer of miR-200a to senescent endothelial cells causing Keap1 down-regulation and subsequent Nrf2 expression, which is a vital pathway in anti-aging processes (Fig. 1) [47]. Moreover, human periodontal ligament stem cells (PDLSCs)-exosomes may have anti-aging impacts through the transfer of miR-141-3p to downregulate KEAP1 expression and consequently activate the Nrf2 antioxidant pathway (Fig. 1) [30].

## Acute kidney injury (AKI)

AKI is a commoncondition marked by a sudden decrease in renal function. Various pathological factors, including I/R, sepsis, trauma and exposure to nephrotoxic substances can trigger AKI [216, 217]. Kidney I/R is one of the most common causes of AKI. After ischemic damage, renal tubular cells, specifically those in the proximal tubules, undergo different forms of cell death [218]. The mortality rate of AKI is disturbingly elevated, ranging from 24 to 62% [219]. Therefore, various therapeutic approaches, including the transplantation of MSC, have been developed. However, due to some limitations in MSC transplantation, some researchers have recommended an alternative approach termed 'cell-free therapy.' This strategy involves the use of EVs derived from stem cells for injury therapy [220]. The protective role of EVs derived from MSCs in kidney injury has been documented [27, 221]; However, the specific mechanism responsible for this protective action is not yet clearly elucidated.

Following I/R injury, oxidative stress leads to ROS accumulation high concentration in renal tubules which have a greater number of mitochondria compared to other structures within the kidney. Excessive amounts of ROS can result in the fragmentation and disturbance of mitochondria in renal cells that triggers the death of renal cells through both necrosis and apoptosis, accompanied by the secretion of pro-apoptotic proteins [222]. However, mitochondria possess defense systems to inhibit the additional ROS generation, namely antioxidant systems, that critically rely on the Keap1 -Nrf2 signaling pathway. It has been documented that MSC-EVs are able to alleviate I/R-induced AKI and contribute to the preservation of redox homeostasis by promoting the activation of Nrf2/ARE signaling [223, 224]. The researchers in this investigation proposed a hypothesis suggesting that the activation of Nrf2 may be attributed to some miRNAs transported by EVs derived from MSC. Another I/R research supports this mechanism in which human placenta-MSC sEVs were found to deliver miR-200a-3p to tubular epithelial cells. The presence of this miR led to the downregulation of Keap1, nuclear translocation of Nrf2 and promotion of SOD2 expression. This antioxidant defense mechanisms resulted in elevated ATP production and shielded tubular epithelial cells against mitochondrial fragmentation (Fig. 1) [225]. It has also been found that sEVs derived from hUC-MSCs modified with angiotensin-converting enzyme (ACE) can hinder apoptosis, diminish oxidative stress and regulate inflammatory responses, leading to the reduction of renal I/R injury. The probable mechanism for this effect may be related to the activation of the Nrf2/HO-1 pathway [226].

## Skin injuries

Skin is the largest organ of the body, serving as a protective agent against environmental toxins and microorganisms as well as preventing dehydration. Additionally, it performs crucial functions like immune surveillance, self-healing and sensory detection. Skin damage often results from acute traumas, infections, chronic wounds, surgical procedures, diabetic ulcers and genetic disorders [227]. As an important paracrine factor secreted from stem cells, exosomes show regenerative functions in a wide range of diseases. Exosomes derived from iPSC-derived MSC are able to improve the wound healing process through enhancing collagen production, angiogenesis and fibroblasts proliferation/migration in human dermatom [228]. Ultraviolet radiation triggers oxidative stress which results in ROS production [229, 230] and causes DNA fragmentation or lipid peroxidation that ultimately results in several skin damages like premature aging, sunburn ore even carcinogenesis [230–232]. As a key component, Nrf2 is involved in the regulation of antioxidant enzymes following skin injury [233] and application of an Nrf2 agonist has been shown to improve mottled hyperpigmentation in the photodamaged skin [234]. These results suggest that regulation of Nrf2 activity can be considered as an effective therapeutic approach for treating skin injury. Activating the Nrf2 pathway internally or systemically in response to skin injury triggers a chain reaction, leading to potent antioxidant production such as SOD, GPX, and catalase (CAT) [233, 235]. Exosomes derived from MSCs could insert skin repair after ultraviolet B radiation-induced ROS production via Nrf2 signaling pathway activation which results in expression of cytoprotective antioxidants and DNA damage inhibition [191, 236].

## Conclusion

We discussed the therapeutic potentials of stem cell‐derived exosomes with a focus on Nrf2 regulatory role and its potential application as a novel cell-free therapy approach for several human diseases (see Table 1). Application of stem cell-derived exosome opens a new window for repairing, regenerating, and treating a range of disorders by stimulating different pathways. Exosomes therapy is able to modulate and re-program cell function through delivery of biomolecules and therapeutic compounds to different target tissues, indicating the substantial potential of these tiny vesicles. Nrf2 signaling pathway plays a key role in the regulation of exosomes therapeutic potential and exerts therapeutic effects against diseases through the activation of antioxidant signaling pathways to protect the cells from the detrimental effects of oxidative stress. Based on the context discussed above, promotion of the antioxidant properties by the Nrf2 activation with stem cell derived exosomes administration can represent a novel focus for future research efforts. Therefore, Nrf2 activation can be considered as promising therapeutic approach, however there is a need to develop suitable approaches and methods to tailor exosomes with high drug loading capacity, increased target specificity and non–cytotoxic effects.

**Table 1:** Studies indicating Nrf2 role in therapeutic effects of exosomes derived from various stem cell sources

Exosome cell source/species	Cargo and Loading mechanism	Exosome markers	Pathologies	Outcomes	Nrf2^−/−^ or Nrf2 inhibitor	Refs.
BMSC/ SD rat	–	CD9, CD63, TSG101	Diabetic wound	Exosomes improved the formation of EPC tubes, sped up the healing process, and reduced inflammation in diabetic wounds. By utilizing a Nrf2 activator, the therapeutic advantages of the exosomes were enhanced by Nrf2 activation	Nrf2 shRNA	[45]
ADSC/SD rat	miR-130a-3p (transfection)	CD9, CD63, TSG101	Diabetic peripheral neuropathy	miR-130a-3p delivery through ADSC-derived EVs activated Nrf2/HIF1α/ ACTA1 pathway by DNMT1 suppression to improve diabetic peripheral neuropathy through inhibiting schwann cells apoptosis	Nrf2 shRNA	[67]
ADSC/ C57BL/KsJ db/m mice	–	CD9, CD63, CD81	Diabetic nephropathy	Exosomes effectively alleviated inflammation and oxidative stress caused by high glucose in podocytes by upregulation of FAM129B and reactivation of the Nrf2-HO-1 pathway	HO-1 siRNA	[60]
hUC-MSC/ Human	circHIPK3 (transfection)	CD63, CD81, TSG101	Diabetes mellitus	circHIPK3 overexpressing exosomes provided a protective effect against cell injuries induced by high glucose and promoted angiogenesis in diabetic wounds by the direct inhibition of miR-20b-5p activity which leads to upregulation of Nrf2 and VEGFA expression	–	[74]
BMSC	circ-ITCH (transfection)	CD63, CD9, TSG101	Diabetic foot ulcers	Exosomal circ-ITCH blocked ferroptosis and stimulated the HUVECs angiogenesis by Nrf2 pathway activation through the recruitment of TAF15 protein and ultimately accelerated the healing process of wound	Nrf2 shRNA	[91]
ADSC/ Human or rat	Nrf2 (transfection)	CD4, CD63, TSG101	Diabetic foot ulcer	Exosomes extracted from Nrf2-overexpressing stem cells improved foot ulceration in diabetic rats	–	[87]
BMSC/ C57BL/6 J mice	–	CD63, TSG101	Respiratory distress syndrome	Exosomes mitigated sepsis-induced AECII apoptosis through restoring the mitochondrial dysfunction mediated by Nrf2	ML385 Nrf2 inhibitor	[140]
ADSC/ Human	miR-125b-5p	CD63, CD9, TSG101	Sepsis lung injury	Delivery miR-125b-5p via exosomes reduced inflammation-induced ferroptosis in PMVECs and protected lung damage by regulation of Keap1/Nrf2/GPX4 axis	–	[133]
hUC-MSC/ Human	miR-199a-5p (transfection)	HSP70, CD63, TSG101	Acute lung injury	Exosomes enhanced the Nrf2 expression and translocation into the cell nucleus by transporting miR-199a-5p and interacting with CAV1 which led to upregulation of antioxidant enzymes expression within lung cells and regulation of oxidative stress	–	[134]
hAMSC/ Human	Nrf2 (transfection)	CD9, CD63, CD81	Acute lung injury	sEVs obtained from Nrf2-overexpressing hAMSCs protected against LPS -induced lung injury through preventing the NLRP3 activation and promoting the M2 macrophages polarization	Nrf2 siRNA	[126]
BMSC/ C57BL/6 mice	circ_0072464 (transfection)	CD63, CD81, TSG101	Intervertebral disc degeneration	circ_0072464 transported by EVs increased the levels of Nrf2 expression by competitive binding with miR-431 which led to ferroptosis suppression in nucleus pulposus cells and IDD alleviation	Nrf2 shRNA	[115]
BMSC/Human	–	CD63, CD81	Intervertebral disc degeneration	Exosomes restored the down-regulated antioxidant response system by modulation of the Keap1/ Nrf2 axis in degenerating nucleus pulposus cells	ML385 Nrf2 inhibitor, Nrf2 siRNA	[114]
ADSC/SD rat	–	CD63, CD81, HSP-70	Dexamethasone-induced bone loss	Exosomes reduced oxidative stress and apoptosis by regulation of Nrf2/HO-1 expressions and prevented the progression of glucocorticoid-induced osteoporosis	Nrf2 siRNA	[11]
hUC-MSC/Human	–	TSG101, CD9, CD63	Non-alcoholic steatohepatitis	Exosomes exerted anti-lipid deposition, anti-oxidative stress and anti-inflammatory effects through activation of Nrf2/NQO-1 pathway	ML385 Nrf2 inhibitor	[185]
hUC-MSC/ Human	miR-24-3p (transfection)	CD9, CD63, CD81	Nonalcoholic fatty liver disease	Delivery of miR-24-3p by exosomes effectively targeted Keap-1, leading to the inhibition of lipid synthesis and NF-kB signaling pathways, and improvement of Nrf2 activation in NAFLD	Keap1 siRNA	[179]
BMSC/ C57BL/6 mice	Baicalin (transfection)	CD9, CD63, CD81, TSG101	Acute liver injury	Exosomes derived from Baicalin-pretreated MSC exhibited a protective effect on liver function and activated the Keap1- Nrf2 pathway by P62, which prevents ROS generation and inhibits ferroptosis induced by lipid peroxides	ML385 Nrf2 inhibitor	[172]
iPSCs–MSC/Human	–	–	Myocardial injury	Exosome led to promoted cardiomyocyte viability, improved cardiac function, reduced infarction ratio, and suppressed oxidation levels by activation Akt/Nrf2/HO-1 pathway	LY294002 PI3K/Akt inhibitor	[159]
BMSC/ C57BL/6 mice	FNDC5 (transfection)	CD63, CD81, ALIX	Myocardial infarction	Exosomes derived from FNDC5-preconditioned BMSCs played a protective role against MI through anti-inflammatory effects and polarization of macrophage which partly reduced NF‐κB and upregulated Nrf2/ HO-1 Axis	SnPP HO-1 inhibitor	[46]
BMSC/SD rat	Nrf2 (transfection)	CD63, CD81, CD9, TSG101, Alix	Atrial fibrillation	Lv-Nrf2 exosomes delivery suppressed arrhythmias induced by atrial fibrillation, myocardial apoptosis, fibrosis and inflammation by the Nrf2/HO-1 axis	–	[24]
ADSC/ SD rat	–	CD9, CD63, HSP70	MTX-induced neuronal damage	Exosomes inhibited oxidative stress triggered by MTX in hippocampus neurons by Nrf2-ARE activation	ML385 Nrf2 inhibitor	[196]
BMSC/Mouse	–	CD63	Delayed neurocognitive recovery (dNCR)	Exosome improved cognitive function by suppressing hippocampus ferroptosis through activation of the SIRT1/Nrf2/HO-1 axis in dNCR aged mice	EX-527 SIRT1 inhibitor	[197]
hUC-MSC/Human	–	Flotillin-1, CD63, TSG101	LPS/H2O2-induced neuroinflammation	Exosomes blocked the Nrf2/NF-κB p65/NLRP3 signaling pathway to attenuate oxidative stress and neuroinflammation, as well as increase a shift microglia phenotype from pro- to anti-inflammatory	ML385 NRF2 inhibitor	[237]
ADSC/C57BL/6 mice	circAkap7 (transfection)	CD9, CD63, TSG101	Cerebral ischemic injury	Exosome-circAkap7 provided protection against ischemic damage through enhancing ATG12-mediated autophagy and alleviated oxidative stress via increasing nuclear transcription of Nrf2 by absorbing miR-155-5p	Nrf2 siRNA	[194]
BMSC/SD rat	miR-194 (transfection)	CD63, TSG101	OGD/R	Exosomes loaded with miR-194 alleviated damage caused by OGD/R by suppressing expression of Bach1 and promoting the Nrf2/HO-1 axis activation via delivery of miR-194 to endothelial cells of brain vessels, which led to the ferroptosis reduction in these cells following hypoxic-ischemic brain injury	–	[193]
hNSC/Human	–	CD9, CD63, CD81	Hypoxia-reperfusion injury	Coculture of human NSCs-derived EVs with neurons inhibits the apoptosis of the neurons by inducing the translocation of NRF2 to neuronal nuclei, regulating the expression of oxidative stress-induced kinases	–	[195]
BMSC/Wistar rat	miR-23b (transfection)	CD81, CD63, TSG101	Intracerebral hemorrhage	Exosomal miR-23b ameliorated oxidative stress and neuroinflammation in ICH. PTEN acts as a target gene of miR- 23b by regulation of the Nrf2 signaling pathway and activation of NLRP3 inflammasome	–	[198]
T-MSCs human	miR-100-5p	TSG101, CD9, HSP70	Parkinson disease	Exosomes enriched with miR-100-5p provide a protective role against the degeneration of dopamine neurons and contribute to the preservation of the function of nigrostriatal system, improvement of motor impairments and mitigation of oxidative stress by modulating the Nox4-ROS-Nrf2 axis	ML385 Nrf2 inhibitor	[199]
hUC-MSC/Human	–	CD63, CD81	Oxidative stress-induced skin injury	Exosomes attenuated oxidative stress-induced skin damage by decreasing ROS production and improving the antioxidant capacities by the Nrf2 defense system regulation	ML385 Nrf2 inhibitor, Nrf2 siRNA	[191]
ADSC/Rat	–	CD9, CD63, CD81	Ultraviolet B-mediated Photoaging	Exosomes prevented ROS generation and DNA damage induced by UVB through activating the Nrf2 pathway and promoting protective antioxidants expression	–	[236]
Placenta- MSC/Human	miR-21 (transfection)	CD63, CD9, HSP70	Aging-related oxidative damage	Exosomes containing miR-21 upregulated the expression of the PTEN/PI3K-Nrf2 axis in senescent CD4 + T cells, improved their antioxidant capabilities, thereby attenuating age-related immunological dysfunction	PTEN inhibitor bpV (HOpic)	[214]
hESC/Human	miR-200a	CD9, CD63, TSG-100	Aged mouse skin pressure ulcer model	Exosomes accelerated wound healing and improved angiogenesis by rejuvenating endothelial senescence. In addition, they exerted the anti-aging impacts through the transfer of miR-200a to senescent endothelial cells and Nrf2 signaling activation	Brusatol Nrf2 inhibitor	[47]
PDLSC/Human	miR-141-3p	CD9, CD63, CD81, TSG101	High glucose induced senescence	Exosomes exhibited anti-aging effects by miR-141- 3p delivery to reduce KEAP1 expression and activate the Nrf2 antioxidant pathway	ML385 Nrf2 inhibitor	[30]
Placenta- MSC/Human	miR-200a-3p	TSG101 ALIX, CD9, CD63	AKI model induced by IRI	miRNA-200a-3p delivered by MSC-EVs activated the Keap1-Nrf2 pathway in TECs to exert antioxidant effects, which helps restore renal function by regulating mitochondrial structure and function	-	[225]
hUC-MSC/Human	–	CD9, CD44, CD63, CD73	AKI model induced by IRI	MSC-sEVs have the potential to alleviate AKI caused by I/R and can assist in balancing oxidative stress and antioxidative responses by enhancing the activation of Nrf2/ARE	–	[223]
hUC-MSC/Human	ACE2 (transfection)	CD9, CD63, TSG101	AKI model induced by IRI	MSC-ACE2-sEVs could protect the kidney against I/R injury, and this effect could be attributed to Nrf2/HO-1 axis activation	–	[226]

## Data Availability

Not applicable.

## Abbreviations

ACTA1: Skeletal muscle actin alpha 1
ADSCs: Adipose-derived stem cells
AECII: Type II alveolar epithelial cell
AKI: Acute kidney injury
Akt: Protein kinase B
ALI: Acute lung injury
ARDS: Acute respiratory distress syndrome
ATG12: Autophagy Related 12
Bach1: CNC homology 1
BMSC: Bone marrow-derived stem cell
CAT: Catalase
Circ-ITCH: CircRNA-itchy E3 ubiquitin protein ligase
CNS: Central nervous system
CXCL12: C–X–C motif chemokine ligand 12
CXCR7: C–X–C chemokine receptor type 7
DFU: Diabetic foot ulcers
D-GaIN/LPS: D-galactosamine and lipopolysaccharide
DNMT1: DNA methyltransferase 1
DPN: Diabetic peripheral neuropathy
ECM: Extracellular matrix
EPCs: Endothelial progenitor cells
EVs: Extracellular vesicles
FNDC5: Fibronectin type III domain-containing protein 5
GCL: Glutamate-cysteine ligase
GPX: Glutathione peroxidase
hAMSC: Human amniotic mesenchymal stem cells
HDFs: Human dermal fibroblasts
hESCs: Human embryonic stem cells
HIF1α: Hypoxia inducible factor 1 subunit alpha
hNSC: Human neural stem cells
HO-1: Heme oxygenase-1
hUC-MSCs: Human umbilical cord mesenchymal stem cells
HUVECs: Human umbilical vein endothelial cells
IDD: Intervertebral disc degeneration
IL-1: Interleukin 1
IL-6: Interleukin 6
iPSCs: Human-induced pluripotent stem cells
I/R: Ischemia–reperfusion
KEAP1: Kelch-like ECH-associated protein 1
LPS: Lipopolysaccharides
MDA: Malondialdehyde
MI: Myocardial infarction
miR: MicroRNAs
MSC*s: Mesenchymal stem cells
MTX: Methotrexate
NAD+ NAFLD: Nicotinamide adenine dinucleotide Non-alcoholic fatty liver disease
NASH: Non-alcoholic steatohepatitis
Nox4: NADPH oxidase 4
NF-κB: Nuclear factor kappa B
NLRP3: NLR family pyrin domain containing 3
NPCs: Neural stem/progenitor cells
NQO1: NAD(P)H quinone oxidoreductase 1
Nrf2: Nuclear factor erythroid 2–related factor 2
OGD/R: Oxygen–glucose deprivation/reoxygenation
PDLSCs: Human periodontal ligament stem cells
PI3K: The phosphoinositide 3-kinase
PMVECs: Pulmonary microvascular
PTEN: Phosphatase and tensin homolog deleted on chromosome 10
ROS: Reactive oxygen species
SASP: Senescence-associated secretory phenotype
sEVs: Small extracellular vesicles
Sirt1: Silent information regulator 2 homolog 1
SOD: Superoxide dismutase
TAF15: TATA-Box-binding protein associated factor 15
tBHQ: Tert-butylhydroquinone
TGF-β: Transforming growth factor beta
T-MSCs: Trophoblast stage-derived MSCs
TNFα: Tumor necrosis factor alpha
UV: Utlraviolet
VEGFA: Vascular endothelial growth factor-A
